# Supplementary material for: GREGoR: Accelerating Genomics for Rare Diseases
Source: ArXiv. 2024 Dec 18:arXiv:2412.14338v1. Preprint. [Version 1] (PMC11702807)
Supplement: 1 [file NIHPP2412.14338v1-supplement-1.pdf]

Supplementary Table 1: Tracking GREGoR Papers With Molecular Diagnoses

Gene	PMID	Functional Work
ACBD6	34582790	No
ACBD6	37951597	Yes
ACCN2	34582790	No
ACOT7	34582790	No
ACTC1	37457373	No
ACTL6A	34582790	No
ACTL6B	39275948	Yes
ACTR1B	39033378	No
ADAM19	34582790	No
ADAMTS15	35962790	Yes
ADSL	34582790	No
AFF3	38811945	Yes
AHDC1	34582790	No
AHDC1	33372375	No
AHDC1	34950897	No
ALS2	34582790	No
AMPD2	34582790	No
ANK3	38988293	No
ANKRD11	34582790	No
AP3B2	34582790	No
AP4B1	34582790	No
APTX	34582790	No
ARAP1	34582790	No
ARFGEF3	38258669	Yes
ARID1B	34582790	No
ARID4A	34582790	No
ARV1	34582790	No
ARX	34582790	No
ASH1L	34582790	No
ASNS	34582790	No
ASPM	34582790	No
ASTN1	34582790	No
ASTN2	34582790	No
ASXL3	34582790	No
ATP1A1	34582790	No
ATP1A3	37043503	Yes
ATP5F1A	34954817	Yes
ATP5F1E	34954817	Yes
ATP5MC3	34954817	Yes
ATP5PO	34954817	Yes
ATP7A	34582790	No
ATRX	34582790	No
BARD1	34582790	No
BHLHA9	36035248	No
BMPER	34582790	No
BRWD3	34582790	No

C2ORF69	34582790	No
CACNA2D2	34582790	No
CAMSAP1	34582790	No
CAPN3	34816580	No
CASP5	37603195	Yes
CBX6	34582790	No
CC2D1B	34582790	No
CCDC39	39606420	No
CCDC40	39606420	No
CCNO	39606420	No
CDK10	34582790	No
CDKL5	35934918	No
CDKL5	34582790	No
CELF2	38258669	Yes
CELSR3	38429302	Yes
CEP290	34582790	No
CEP85L	34582790	No
CFAP46	39606420	No
CFAP47	38633811	Yes
CHASERR	39442041	Yes
CHD2	39442041	Yes
CHD3	34582790	No
CHMP1A	34582790	No
CIT	34582790	No
CLP1	34582790	No
CNTN5	34582790	No
CNTNAP2	34582790	No
COBL	34582790	No
COG3	37711075	Yes
COL6A1	38585825	Yes
COPB1	34582790	No
CREB3	34582790	No
CRYBB2	38990107	No
CTBP1	34582790	No
CTNNA1	38585811	No
CUX1	38585811	No
CYP1B1	38990107	No
DCX	34582790	No
DDC	34582790	No
DDX3X	34582790	No
DEAF1	34582790	No
DENND5A	39174524	Yes
DHCR24	34582790	No
DHX9	37467750	Yes
DLGAP1	34582790	No
DNAAF2	39606420	No
DNAAF4	39606420	No
DNAH1	39606420	No

DNAH1	39606420	No
DNAH11	39606420	No
DNAH5	39606420	No
DNAH6	39606420	No
DNAH6	39606420	No
DNAH8	39606420	No
DNAH9	39606420	No
DNAJC8	34582790	No
DPF2	34582790	No
DRC1	39606420	No
DUSP4	34582790	No
DYRK1A	34582790	No
EEF1A2	34582790	No
ELF4	36477361	Yes
ENPP6	34582790	No
ENTPD1	35471564	Yes
EPG5	34582790	No
EPHA8	34582790	No
ERCC6	34582790	No
ESAM	34582790	No
ESAM	36996813	Yes
EXOSC3	34582790	No
FA2H	34582790	No
FAM120A	34582790	No
FAM91A1	34582790	No
FBP2	38258669	Yes
FBXW11	34582790	No
FER	38585811	No
FGF21	38585811	No
FLNA	39606420	No
FLVCR1	39306721	Yes
FOXG1	34582790	No
FOXI3	37041148	Yes
FOXN4	34582790	No
FRMD7	34582790	No
FSHR	34582790	No
GAS8	39606420	No
GATSL3	34582790	No
GCC2	34582790	No
GCH1	35083481	No
GGPS1	35869884	No
GIN1	34582790	No
GIPR	34582790	No
GIT1	34582790	No
GJC2	34582790	No
GLB1	34582790	No
GLI2	38990107	No
GLI2	34582790	No

GLI3	36035248	No
GMPPB	34582790	No
GNAS	35811283	No
GNAS	38585811	No
GOLGA2	34582790	No
GOLGA4	34582790	No
GPR87	36622818	No
GPT2	34582790	No
GREB1L	37124138	No
GRM7	34582790	No
HECTD3	34582790	No
HECTD4	34582790	No
HECTD4	36401616	No
HEXB	34582790	No
HMGCR	37167966	Yes
HOXD	36035248	No
HOXD13	36035248	No
HPDL	34582790	No
HPS1	34582790	No
HSPB1	33686258	No
HYAL2	34906488	Yes
HYDIN	39606420	No
ITGB8	34582790	No
JRK	34582790	No
KANSL3	38258669	Yes
KCNJ14	34582790	No
KCTD7	34582790	No
KDM2B	34582790	No
KDM5A	34582790	No
KDM5B	39202393	No
KDM5C	34582790	No
KIAA0430	34582790	No
KIF1A	34582790	No
KIF21A	38585811	No
KIF26A	34582790	No
KIF26A	36228617	Yes
KIF5C	38585811	No
KIF7	39606420	No
KIFC3	34582790	No
KLB	38585811	No
L1CAM	34582790	No
LAMA1	34582790	No
LAMB3	34582790	No
LAMC3	34582790	No
LARGE1	34582790	No
LARP7	34582790	No
LCTL	34582790	No
LGI3	35948005	Yes

LPAR6	34582790	No
LRP2	34582790	No
LSS	37157980	No
MAP2K4	38258669	Yes
MAP3K20	38451290	No
MAP3K7	34582790	No
MCM3AP	34582790	No
MCM6	38258669	Yes
MCPH1	34582790	No
MDM1	38868186	Yes
MEGF8	39606420	No
MGAT2	34582790	No
MGP	37923733	Yes
MKS1	34582790	No
MPZ	38585811	No
MRPS25	34582790	No
MRPS25	39606420	No
MRPS27	34582790	No
MTOR	34582790	No
MTSS2	36067766	Yes
MUSK	34816580	No
MYH1	34582790	No
MYH10	38585811	No
NALCN	34582790	No
NANS	34582790	No
NAV2	34582790	No
NES	38585811	No
NETO1	36622818	No
NFE2L3	38258669	Yes
NGEF	34582790	No
NGLY1	34582790	No
NHLRC2	37188825	Yes
NLK	34582790	No
NODAL	38570875	No
NOTCH1	33686258	No
NPHP3	39606420	No
NPR2	36035248	No
NRD1	34582790	No
NSD1	34582790	No
NTNG2	34582790	No
NUAK1	34582790	No
OCLN	34582790	No
OLIG2	38585811	No
OTOA	33492714	No
OTUD6B	35430327	No
PAFAH1B1	34582790	No
PARD3B	34582790	No
PAX5	35094443	No

PCDH18	34582790	No
PDK1L1	39606420	No
PDZD2	34582790	No
PEX6	34582790	No
PGAP3	34582790	No
PHF8	35469323	No
PIK3C2A	34582790	No
PKD1	34582790	No
PLAA	34582790	No
PLCG1	38260438	Yes
PLD3	34582790	No
PLK4	34582790	No
PLXNA1	34582790	No
PNKP	34582790	No
POLR1D	34582790	No
POLR3A	34582790	No
POMGNT1	34582790	No
PPP1R15A	34582790	No
PPP1R21	38356149	No
PPP1R35	34582790	No
PPP1R35	36598158	Yes
PPP1R3F	37531237	Yes
PPP2R1A	34582790	No
PPP2R5C	38258669	Yes
PREX2	34582790	No
PTCHD2	34582790	No
PYCR2	34582790	No
RAC3	35851598	Yes
RAD21	34582790	No
RANBP3L	34582790	No
RARB	37092537	Yes
RASGRF2	34582790	No
RBM10	34582790	No
RCOR3	34582790	No
RNASEH2A	34582790	No
RNASEH2B	34582790	No
RNU4-2	38991538	No
ROBO1	35227688	Yes
ROBO3	38585811	No
ROBO3	34582790	No
RPA1	34582790	No
RSPH4A	39606420	No
RSPH4A	39606420	No
RSPO4	34582790	No
RTN2	34582790	No
RXRA	38990107	No
SCN1A	34582790	No
SCN7A	34582790	No

SEMA3F	38585811	No
SERAC1	34582790	No
SERPINB8	36622818	No
SETX	34582790	No
SF3B1	38258669	Yes
SHANK3	34582790	No
SHROOM4	34582790	No
SHROOOM3	39606420	No
SLC12A5	38585811	No
SLC18A2	34582790	No
SLC19A3	34582790	No
SLC25A45	34582790	No
SLC30A7	35751429	No
SLC30A7	35751429	No
SLC37A1	34582790	No
SLC39A10	34582790	No
SLC4A10	38054405	Yes
SLC5A7	39135055	No
SLC6A1	34582790	No
SLC7A1	34582790	No
SMARCA1	34582790	No
SMC3	38297832	No
SMPD1	34582790	No
SNAPC4	36965478	Yes
SNX14	34582790	No
SORCS2	34582790	No
SOX11	34582790	No
SPAST	36103453	No
SPR	34582790	No
SRD5A3	34582790	No
SRSF1	34582790	No
SSH3	34582790	No
SSTR3	34582790	No
STOML1	34582790	No
STON2	34582790	No
STRTS locus	38714869	Yes
STUB1	34582790	No
SUCLA2	34582790	No
SUFU	34675124	No
SYDE1	36622818	No
SYNE1	38716726	Yes
SYNGAP1	34582790	No
TALDO1	34677006	No
TBC1D23	34582790	No
TBX6	31888956	Yes
TCEAL1	36368327	Yes
TERF2	34582790	No
TGFBR2	38585811	No

THOC6	34582790	No
TLK1	38868186	Yes
TLR7	35477763	Yes
TMOD1	34582790	No
TNRC6B	34582790	No
TOR1A	36757831	No
TPH2	34582790	No
TRAK1	34582790	No
TRAPPC4	34582790	No
TRIM66	34582790	No
TRIT1	34582790	No
TRMT1	34582790	No
TRMT2B	34582790	No
TTC12	39606420	No
TTLL11	34582790	No
TUBA1A	34582790	No
TUBB	38585811	No
TUBB4A	38585811	No
TUBB4A	34582790	No
TUBB6	34582790	No
TUBGCP2	34582790	No
UBE3C	36401616	No
UBR5	38258669	Yes
ULK2	34582790	No
UPS54	34582790	No
VANGL1	38669183	Yes
VANGL2	38669183	Yes
VPS28	34582790	No
VRK3	34582790	No
VSTM2L	34582790	No
WARS2	34582790	No
WDR45B	35322404	No
WDR62	34582790	No
WDR7	34582790	No
WDR73	34582790	No
WDR81	34582790	No
WDR83OS	34582790	No
WNT10B	36035248	No
WRN	35534204	No
WSB1	34582790	No
XRN1	38258669	Yes
YWHAG	38491959	No
ZBTB34	38258669	Yes
ZC4H2	34816580	No
ZFHX3	38412861	Yes
ZFHX4	39148819	Yes
ZNF462	38585811	No
ZRSR2	34582790	No
